# The Effect of A Geriatric Assessment on Treatment Decisions for Patients with Lung Cancer

**DOI:** 10.1007/s00408-017-9983-7

**Published:** 2017-03-09

**Authors:** Karlijn J. G. Schulkes, Esteban T. D. Souwer, Marije E. Hamaker, Henk Codrington, Simone van der Sar-van der Brugge, Jan-Willem J. Lammers, Johanneke E. A. Portielje, Leontine J. R. van Elden, Frederiek van den Bos

**Affiliations:** 1 Department of Internal Medicine, Diakonessenhuis Utrecht/Zeist/Doorn, Bosboomstraat 1 3582 KE Utrecht, The Netherlands; 2 Department of Internal Medicine, Haga Hospital, Leyweg 275 2545 CH The Hague, The Netherlands; 3 Department of Geriatric Medicine, Diakonessenhuis Utrecht, Utrecht, The Netherlands; 4 Department of Pulmonology, Haga Hospital, The Hague, The Netherlands; 5 Department of Pulmonology, Amphia Hospital, Molengracht 21 4818 CK Breda, The Netherlands; 6 Department of Pulmonology, UMC Utrecht, Heidelberglaan 100 3584 CX Utrecht, The Netherlands; 7 Department of Pulmonology, Diakonessenhuis Utrecht, Utrecht, The Netherlands

**Keywords:** CGA, Frailty, Geriatric assessment, Pulmonary malignancies

## Abstract

**Background:**

Decision-making for older patients with lung cancer can be complex and challenging. A geriatric assessment (GA) may be helpful and is increasingly being used since 2005 when SIOG advised to incorporate this in standard work-up for the elderly with cancer. Our aim was to evaluate the value of a geriatric assessment in decision-making for patients with lung cancer.

**Methods:**

Between January 2014 and April 2016, data on patients with lung cancer from two teaching hospitals in the Netherlands were entered in a prospective database. Outcome of geriatric assessment, non-oncologic interventions, and suggested adaptations of oncologic treatment proposals were evaluated.

**Results:**

83 patients (median age 79 years) were analyzed with a geriatric assessment, of which 59% were treated with a curative intent. Half of the patients were classified as ECOG PS 0 or 1. The majority of the patients (78%) suffered from geriatric impairments and 43% (*n* = 35) of the patients suffered from three or more geriatric impairments (out of eight analyzed domains). Nutritional status was most frequently impaired (52%). Previously undiagnosed impairments were identified in 58% of the patients, and non-oncologic interventions were advised for 43%. For 33% of patients, adaptations of the oncologic treatment were proposed. Patients with higher number of geriatric impairments more often were advised a reduced or less intensive treatment (*p* < 0.001).

**Conclusion:**

A geriatric assessment uncovers previously unknown health impairments and provides important guidance for tailored treatment decisions in patients with lung cancer. More research on GA-stratified treatment decisions is needed.

## Introduction

In the Netherlands, over 12,000 new cases of lung cancer are diagnosed every year [1]. Lung cancer is predominantly a disease of the elderly: half of all newly diagnosed patients are over 70 years old [1]. Lung cancer usually shows an aggressive course of disease, and mortality rates are high. It is the leading course of cancer mortality worldwide [2]. Survival rates are even worse in elderly patients (>75), with 1 and 5 year survival rates of 33 and 10%, respectively [1].

Older patients represent a heterogeneous population due to differences in physiological reserves, comorbidity, functional capacity, and the presence of geriatric impairments [3]. As a result of these differences, benefit from lung cancer treatment varies [4–6]. In addition, complications of therapy are common and are more likely to occur in patients with decreased physiological reserves [7].

Currently used measures for quantifying health status and reserves in patients with lung cancer, such as performance status or pulmonary function testing, do not appear to differentiate sufficiently within the elderly population [3]. Even in patients with good performance status, geriatric impairments can be present because impairments in cognitive functioning, depressive symptoms, and malnutrition are easy to miss [7–9].

Therefore, in 2005, a task force of the International Society of Geriatric Oncology (SIOG) recommended that a geriatric assessment should be used to detect these unaddressed problems, improve functional status, and possibly survival [3]. This systematic procedure can be used to objectively appraise the health status, focusing on somatic, functional, and psychosocial domains [3, 10].

Although a myriad of publications have propagated its use, the actual implementation of geriatric assessments in clinical practice has thus far been limited [3, 11–13]. In the Diakonessenhuis and Haga hospital, two large teaching hospitals in the Netherlands, geriatric assessments for patients with lung cancer have been implemented in the standard care for patients over 70 years of age since 2014. In this analysis, we have assessed the yield of this assessment and its effect on treatment decisions.

## Methods

Between January 2014 and April 2016, all consecutive patients with lung cancer aged 70 years and older referred for a geriatric assessment at the Haga hospital in the Hague were included in a prospective database for quality control purposes. No patients were excluded for this initial database. Selection of patients for a geriatric assessment was done if the patient was considered to be potentially frail based on the Geriatric8 (G8) [14] and identification of seniors at risk (ISAR-HP) [15] screening tools or by the referring physician/thoracic oncologist based on clinical judgment. The maximum score of the G8 is 17 points, with a score of 14 or less being defined as impaired [14]. The maximum score of the ISAR-HP is 4, and a score of 2 or more is defined as impaired [15]. Oncologic treatment options were formulated by the thoracic oncologist, based on a complete oncologic work-up, prior to referral for the geriatric assessment.

The geriatric consultations and assessments were performed by three geriatricians specialized in geriatric oncology. Patients were seen together with their family or caregivers if possible. The geriatric assessment was partly performed by a specialized nurse and included an evaluation across eight geriatric domains: comorbid diseases, medication use, diagnosis and, if applicable, treatment of cognitive impairments, mood disorders, nutritional status, functional impairments (mobility, basic, and (instrumental) activities of daily living ((I)ADL)), and social network or supportive care status. Specific geriatric tools per geriatric domain were used on indication: Charlson Comorbidity Index [16] to score comorbidity (a score of ≥2 was defined as impaired), medication use was defined as an impaired geriatric domain if patients used three or more drugs or in case of inappropriate prescription, mini nutritional assessment (maximum 27 points, impaired ≤ 23) [17], mini mental state examination (maximum 30 points, impaired ≤ 23.5) [18], geriatric depression scale (maximum 15 points, possible depression ≥6) [19], timed-up-and-go-test (impaired ≥ 12 s) [20, 21], hand grip strength (age-related cutoff values, no adjustment from the original research) [22], Katz index (six items scored, impaired ≥ 2) [23], and Lawton (maximum 8 points, 0 indicating fully dependency, impaired ≥ 2) [24] were used for scoring ADL en IADL, respectively. The geriatrician interpreted the assessment outcomes, reflected on them with patient and caregivers, proposed interventions for optimization impairments that were found, and discussed the patients’ preferences and expectations.

Based on this assessment and consultation, the geriatrician evaluated the patient’s capacity to tolerate treatment within the multidisciplinary lung cancer team and if needed, proposed an adaptation of oncologic treatment, tailored to the patient’s capacities, health limitations, and preferences. If applicable, advanced care planning was initiated.

The treatment adaptations were labeled as ‘no change’ if the geriatrician agreed with the treatment plan of the oncologist. If the geriatrician advised for a different regimen than suggested by the oncologist, these changes were categorized as ‘more intensive’ or ‘less intensive.’

### Data Collection

The regional ethics committee and institutional review board of both hospitals approved this study. The primary endpoint was the effect of the geriatric assessment on (adaptation of) oncologic and non-oncologic treatment decisions. Secondary endpoints were the prevalence of geriatric impairments, the incidence of newly diagnosed geriatric syndromes or medical conditions, and the additional yield of the assessment in terms of advanced care planning, managing the patients’ expectations, and clarifying the patients’ priorities and preferences.

The following data were collected from the medical record: patient demographics (age, sex, Eastern Cooperative Oncology Group Performance Status (PS) [25], comorbidity measured by the Charlson comorbidity index (CCI) [16]), tumor data (tumor type, staging), initial oncologic treatment plan and alternative options prior to geriatric assessment, final oncologic treatment following geriatric assessment. In addition, we collected information on outcome of the geriatric assessment: prevalence of geriatric impairments, incidence of newly diagnosed medical conditions, non-oncologic interventions, suggestions regarding oncologic treatment choices, discussions on advanced care planning, clarification of patients’ priorities, and expectations regarding oncologic treatment.

### Statistical Analysis

For the analysis of our primary outcome, treatment decisions following geriatric assessment were classified as follows: no change, intensified oncologic treatment, less intensive treatment, or supportive care only. Numbers are presented as medians with interquartile ranges (IQR) if not normally distributed. Statistical analyses were performed using SPSS 24.0 (SPSS, Inc., Chicago, IL, USA). A *p* value <0.05 was considered statistically significant. The Chi-square test was used to compare categorical variables between groups.

## Results

### Patient Characteristics

Eighty-three patients were included in the present analysis. Patient demographics can be found in Table 1. The median age of the patients was 79 years (IQR 74–82 years) and 65% were male (*n* = 54). The CCI was 0 or 1 for 23 patients (28%), the remaining 73% (*n* = 60) had a CCI of 2 or higher. The majority of the patients (*n* = 49, 59%) were diagnosed with non-small cell lung cancer (NSCLC), nine patients (11%) were diagnosed with small cell lung cancer (SCLC), two patients (2%) were diagnosed with mesothelioma, and for 23 patients (28%) no histological diagnosis was obtained. Most patients had options for treatment with a curative intent (*n* = 49, 59%), for the remaining patients the treatment intent was only palliative at time of diagnosis and assessment. For 25 patients (30%) the PS was unknown; of the remaining patients 42 were (72%) classified as PS 0 or 1; 11 (19%) patients had a PS of two; and five (9%) patients had a PS of three.

**Table 1:** Patient characteristics

		Total (*n* = 83)
Male (%)		54 (65)
Median age in years (IQR25-75^a^)		79 (74–82)
Diagnosis (%)		
	NSCLC^b^	49 (59)
	SCLC^b^	9 (11)
	Mesothelioma	2 (2)
	No histological diagnosis	23 (28)
Disease stage (%)	I	22 (27)
	II	10 (12)
	III	15 (18)
	IV	22 (27)
	Unknown	14 (17)
Curative treatment options (%)		49 (59)
Charlson comorbidity index (%)	0 or 1	23 (28)
	≥2	60 (72)
ECOG PS^c^ (%)	0	14 (17)
	1	28 (34)
	2	11 (13)
	3	5 (6)
	Unknown	25 (30)

^a^ *IQR25-75* Interquartile ranges 25th and 75th percentile

^b^ *ECOG PS* Eastern Cooperative Oncology Group Performance Status

^c^ *(N)SCLC* non-small cell lung cancer

### Geriatric Assessment

The majority of the patients (*n* = 66, 80%) were referred for a geriatric assessment after risk identification by using Geriatric8 (G8 ≤ 14) or identification of seniors at risk (ISAR-HP ≥ 2), and the remaining 17 patients (20%) were referred by the treating physician based on clinical judgment. For all patients, the GA was performed prior to initiation of oncologic treatment.

Results of geriatric assessments are depicted in Table 2. The majority of the patients (78%; *n* = 65) suffered from one or more geriatric impairments: in 43% (*n* = 35) ≥3 geriatric impairments were identified. Nutritional status was most frequently impaired (52%; *n* = 43), followed by mobility (39%; *n* = 32) and cognitive function (34%; *n* = 28). For 58% of the patients (*n* = 48), the geriatric assessment revealed previously unknown geriatric impairments. Non-oncologic interventions aimed to optimize health status before and during cancer treatment were proposed for 36 patients (43%). Domains that were most frequently amenable for intervention were nutritional status (25%; *n* = 21), followed by impaired mobility based on an impaired timed-up-and-go or low handgrip strength (14%; *n*  = 12) and care dependency in IADL (10%; *n*  =  8). A total of five patients had an impaired GDS and three were subsequently referred for further counseling. Other suggested non-oncologic interventions are described in detail in the Appendix 1 [26].

**Table 2:** Outcome of geriatric assessment

	Prevalence of geriatric impairments	Suggestion for non-oncologic interventions
(Risk of) malnutrition	43 (52%)	21 (25%)
Impaired mobility	32 (39%)	12 (15%)
Cognitive impairments	28 (34%)	6 (7%)
Care dependence in IADL^a^	26 (31%)	8 (10%)
Comorbidity	26 (31%)	4 (5%)
Insufficient social network	20 (24%)	6 (7%)
Care dependence in ADL^a^	17	6 (7%)
Medication issues	9 (11%)	1 (1%)
Psychological issues^b^	5 (6%)	3 (7%)

^a^ *(I)ADL* (Instrumental) activities of daily living

^b^Impaired score on geriatric depression scale

In addition, for 69% (*n* = 57) of patients, the geriatric assessment aided in clarifying patients preferences and expectations or initiating advance care planning.

### Treatment Decisions

**Fig. 1:**
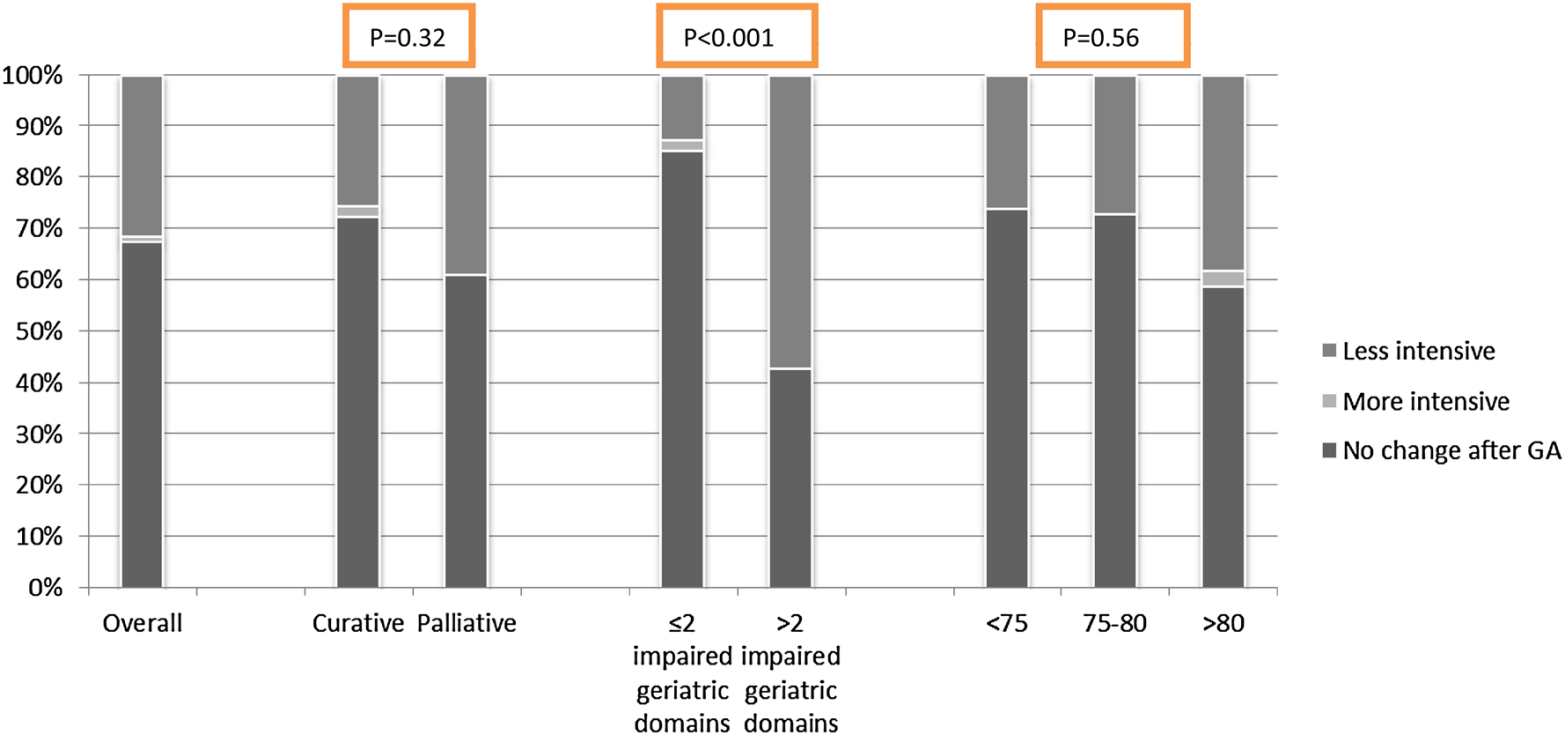
Oncologic treatment suggestions based on geriatric assessment. *Less intensive* the geriatrician advised for a less intensive treatment than suggested by the oncologist, *More intensive* the geriatrician advised for a more intensive treatment than suggested by the oncologist, *No change after GA* there was no difference in oncologic treatment after the geriatric assessment

Based on the geriatric assessment, suggestions for change of the oncologic treatment were proposed in 27 out of 83 patients (33%); the thoracic oncologists adopted all suggestions. These results are shown in Fig. 1 and Appendix 2. A more intensive treatment regimen was suggested for one patient (1%): the geriatrician advised for stereotactic radiotherapy (SBRT) instead of the suggested best supportive care (BSC) of the oncologist. A less intensive treatment regimen was suggested for twenty-six patients (31%). A less intensive treatment suggestion included SBRT instead of surgical resection (*n* = 6) or BSC instead of palliative chemotherapy (*n* = 11), chemoradiotherapy (*n* = 5), or surgical resection (*n* = 4).

We did not find a significant difference in change of treatment based on the geriatric assessment between patients treated with a palliative or curative intent.

For patients with a higher number of geriatric impairments, more often an adapted treatment plan was advised: a less intensive treatment was suggested for 13% of patients (*n* = 6) with ≤2 geriatric impairments versus 57% (*n* = 20) for the patients with >2 geriatric impairments (*p* < 0.001).

No significant difference could be observed by analyzing treatment decisions comparing different age categories (<75, 75–80 and older than 80 years) (*p* = 0.56).

## Discussion

This study shows the results of geriatric assessments and consultations in patients with lung cancer in two teaching hospitals in the Netherlands. The prevalence and number of geriatric impairments was high in the investigated elderly population (78%), especially considering that half of the patients had an ECOG PS of 0 or 1. The geriatric assessment identified previously undiagnosed impairments in 58% of the patients and non-oncologic interventions were advised for 43%. Nutritional status was most frequently impaired, followed by impairments in mobility and cognitive function. For 34% of the patients, adaptations in the oncologic treatment were suggested after the geriatric assessment. With increasing numbers of observed geriatric impairments, less aggressive treatment was more often advised. In addition, the geriatric assessment was often used as a moment to start discussions about preferences and expectations of treatment or initiating advance care planning.

This analysis has several limitations. First, in this type of observational cohort study, a direct comparison of survival and oncologic outcomes between groups is hampered by selection bias and confounding by indication. This could subsequently mean that differences in outcome are incorrectly attributed to the treatment decision, rather than to confounding factors such as poor general health, which affects both treatment choice and outcome. We have no data on health status or treatment decisions in older patients who were not referred. Second, we only reported on the alteration in treatment, but limited data were available about follow-up of how patients subsequently fared. Furthermore, as no control group was available, we were unable to ascertain whether the changes made for the treatment plan resulted in overall better outcomes. Despite these limitations, this analysis provides insight in current clinical practice and the variety of elderly patients with lung cancer that are being referred for a geriatric assessment.

Our findings are in line with prior research that emphasized the importance of a geriatric assessment in the care of elderly patients with cancer [3, 10]. A study among 49 patients with lung cancer in France also showed a high number (45%) of modifications of treatment decisions after a geriatric assessment [27]. Another study, performed in Belgium reported the presence of one or more geriatric impairments in 71% of patients with lung cancer [28]. In a Dutch study among patients with various cancer types, previously undiagnosed impairments were identified in 49% and non-oncologic interventions were initiated in 56% [29].

Our study demonstrates that geriatric assessment can be helpful in the complex decision-making process for elderly patients with lung cancer. Decisions in this heterogeneous population can be complex, particularly because evidence regarding treatment of frail patients is scarce as the patients are frequently excluded from participation in clinical trials [30]. As was previously demonstrated, study results are primarily valid within a population that is comparable to the trial population and do not provide reliable evidence on what the effect would be in other patient groups [31]. As a result, treatment decisions for the elderly will mainly depend on opinions and preconceptions of individual oncologists.

The effect of GA-stratified treatment allocation has not been extensively investigated. A GA-stratified treatment allocation in patients with lung cancer did not improve efficacy but showed comparable survival and appeared to be able to decrease overall toxicity and aggressiveness of treatment [32]. Experiencing less all grade toxicity and receiving less aggressive treatment without losing efficacy can be seen as an important argument to advocate treatment allocation on the basis of a geriatric assessment. More research is urgently needed to further extent these findings.

The incorporation of a routine geriatric assessment in standard oncologic care for all elderly patients with cancer is currently hampered by the time- and resource-consuming nature of these assessments [12, 13]. Furthermore, while there is general consensus that they can be beneficial, there is no clear guideline on when, how, and by whom they should be performed [12, 13]. The presented method of geriatric screening followed by full geriatric consultation and assessment for selected patients may be adequately time efficient. Importantly, it is still a matter of debate whether cancer specialists themselves should take more time to assess patients across multiple (geriatric) domains instead of introducing geriatric consultation by a geriatrician into the care pathway of older patients with cancer, keeping in mind that the latter requires geriatricians with specific expertise in oncology.

An important yield of the geriatric assessment was clarifying patient’s priorities and expectations concerning the proposed treatment options. It appears that this is mostly due to a greater amount of time available for the assessment and does not necessarily require expertise specific to the geriatrician [13]. In an age where the amount of time spent on staging and exploring disease characteristics is rapidly increasing, and more and more money is spent on increasingly sophisticated anti-cancer treatments, taking the time to sit down with a patient and explore what they want and whether or not they will be able to benefit from and tolerate cancer treatment should not be a matter of discussion [33]. However, this will require the incorporation of more elaborate training in the specific needs of frail elderly patients in oncologic study curricula.

## Conclusion

This analysis shows that a geriatric assessment can aid in tailoring treatment decisions, by identifying previously unknown geriatric impairments. Our findings are in line with the SIOG advise that a geriatric assessment should be used in the evaluation of elderly patients with cancer [11]. There is a significant relation between the number of geriatric impairments and the advice for less aggressive treatment. A geriatric assessment is often used as moment to start discussions about preferences and expectations of treatment. Collaboration between geriatricians and oncologists is required to optimize treatment for patients with cancer [29]. More research on GA-stratified treatment decisions in patients with lung cancer is needed.

## Appendix 1

See Table 3.

**Table 3:** Examples of suggested non-oncologic interventions

	Examples of suggested non-oncologic interventions [26]
(Risk of) malnutrition	Referral to dietician, supplemental nutrition drinks
Impaired mobility	Home care, referral occupational therapist, physiotherapist
Cognitive impairments	Home care, start medication, update medication list, referral to specialized nurses
Care dependence in (I)ADL^a^	Home care, occupational therapist, physical therapist
Comorbidity	Update medication list, diagnose and treat comorbidities
Insufficient social network	Home care, specialized nurses, consulting general practitioner
Medication issues	Update medication list
Psychological issues	Referral to general practitioner, referral to psychologist

^a^ *(I)ADL* (Instrumental) activities of daily living

## Appendix 2

See Table 4.

**Table 4:** Change in oncologic treatment after geriatric consultation

Advise oncologist	Advise geriatrician	Number of patients
More intensive		
Best supportive care	SBRT^a^	1
Less intensive		
SBRT^a^	Surgical resection	6
Palliative chemotherapy	Best supportive care	11
Chemoradiotherapy	Best supportive care	5
Surgical resection	Best supportive care	4

^a^ *SBRT* Stereotactic body radiotherapy

## Abbreviations

BSC: Best supportive care
CCI: Charlson comorbidity index
G8: Geriatric 8
(I)ADL: (Instrumental) Activities of daily living
ISAR-HP: Identification of seniors at risk-hospitalized patients
NSCLC: Non-small cell lung cancer
PS: Eastern Cooperative Oncology Group Performance Status
SCLC: Small cell lung cancer
SBRT: Stereotactic body radiotherapy
SIOG: International Society of Geriatric Oncology
